# Trends in the use of health services and their relationship with multimorbidity in Brazil, 1998–2013

**DOI:** 10.1186/s12913-020-05938-4

**Published:** 2020-11-25

**Authors:** Ana Sara Semeão de Souza, José Ueleres Braga

**Affiliations:** 1 Programa de Pós-graduação em Saúde Coletiva, Instituto de Medicina Social, Universidade do Estado do Rio de Janeiro, Rio de Janeiro, RJ Brazil; 2 Departamento de Epidemiologia, Fundação Oswaldo Cruz, Escola Nacional de Saúde Pública Sergio Arouca, Rio de Janeiro, RJ Brazil; 3 Departamento de Epidemiologia, Instituto de Medicina Social, Universidade do Estado do Rio de Janeiro, Rio de Janeiro, RJ Brazil

**Keywords:** Multimorbidity, Health services research, Chronic disease, Multiple chronic conditions

## Abstract

**Background:**

Chronic non-communicable diseases (NCDs) are the leading cause of multimorbidity. Access to effective and equitable health services that meet NCDs’ needs is still limited in many countries. This constitutes the main barrier to coping with NCDs, especially in minimising the suffering of those who are already sick. The present study aimed to identify the relationship between multimorbidity and the use of different health services in Brazil from 1998 to 2013.

**Methods:**

This is a panel study using data from the health supplement of the National Household Sample Survey of 1998, 2003 and 2008 and data from the National Health Survey carried out in 2013. Three health service utilization outcomes were considered: 1. search for health services in the last 15 days (excluding dental services), 2. medical consultation in the previous 12 months and 3. hospitalisations over the last 12 months. Multimorbidity was assessed by counting the number of morbidities from a list of 10 morbidities. Poisson regression models stratified by sex were used to estimate the crude and adjusted prevalence ratios and their respective 95% confidence intervals for each outcome of health service use and multimorbidity, per year.

**Results:**

There was an increase in the prevalence of demand for health services and medical consultations in the last 12 months between 1998 and 2013, regardless of the multimorbidity classification. The prevalence of hospitalisations has decreased over the study period and increased twofold in individuals with multimorbidity. Having multimorbidity increased the use of health services for the three outcomes under the study, being more expressive among men.

**Conclusions:**

This study found that individuals with multimorbidity have higher levels of use of health services. Better understand the multimorbidity epidemiology and the associated impacts on the use and costs of health services can increase the quality of care provided to these patients and reduce rising health care costs.

**Supplementary Information:**

The online version contains supplementary material available at 10.1186/s12913-020-05938-4.

## Background

Chronic non-communicable diseases (NCDs) are the leading cause of morbidity and mortality worldwide and approximately three-quarters of deaths due to NCDs occur in low and middle-income countries [1–3]. A challenge for reducing the disease burden of NCDs, multimorbidity is defined as the coexistence of two or more chronic conditions in the same individual [4]. A recent systematic review estimated the combined global prevalence of multimorbidity to be 33.1%. In high-income countries, the prevalence of multimorbidity was 37.9% and for low and middle-income countries, 29.7% [5].

In Brazil, the prevalence of multimorbidity was estimated at 24.2% in 2013; it was more frequent in women, individuals with less education and at older ages [6]. Besides, it was observed that in Brazil, people develop morbidity and multimorbidity at a younger age than people living in richer countries and women, 10 years earlier compared to men [6–8].

Recent studies have shown that multimorbidity is associated with increased disability and functional decline, reduced well-being and quality of life, and disproportionately higher levels of use of health services with high costs out-of-pocket [9–13]. Currently, the main health care model is focused on the disease rather than the person, therefore, the participation of different caregivers in the management of multiple conditions is inevitable and often results in competing treatments, ill coordination, and inefficient communication between patients and providers, and even unnecessary replication of diagnostic tests or treatments [14–16].

According to the World Health Organization, access to effective and equitable health services that meet the needs of people with NCDs is still limited in many countries. This constitutes the main barrier to coping with NCDs, especially in minimizing the suffering of those who are already sick [17]. Regarding the determinants of the use of health services, it is known that health needs or the existence of the disease, as well as severity and urgency, are proximal factors of use [18].

Despite the high prevalence of multimorbidity in Brazil, only few published studies have examined its relationship with health services [19–21], and none of these studies characterized the patterns of use of health services in individuals with multimorbidity amongst the Brazilian population. With the increasing demand for health systems, it becomes more necessary to identify the profile of individuals with multimorbidity who need health care more urgently, thus aiming to allocate these resources in the most efficient way possible. In this sense, the present study aimed to identify the relationship between multimorbidity and the use of different health services in Brazil, according to their characteristics from 1998 to 2013.

## Methods

### Study design and population

This was a panel study using data from the health supplement of the Brazilian National Household Sample Survey (PNAD) of 1998, 2003 and 2008 and data from the Brazilian National Health Survey (PNS) conducted in 2013. These surveys were carried out by the Brazilian Institute Geography and Statistics (IBGE) in collaboration with the Ministry of Health. Both studies were based on a probabilistic sample of households obtained in three stages of selection and are representative of the Brazilian population. In 1998, 2003, 2008 and 2013, 344,975, 384,834, 391,868 and 205,546 people participated in the survey, respectively. For this work, the study population consisted of adults aged 18 years or over, totaling 889,941 people.

### Study variables

All variables in this study were extracted from the databases of national surveys collected with the same question or an equivalent question repeated in all years. Three outcomes for the use of health services were considered: 1. search for health services in the last 15 days (excluding dental services), 2. medical consultation in the last 12 months and 3. hospitalisations in the last 12 months. The search for services and hospitalisations were assessed dichotomously (yes/no).

Medical visits in the last 12 months for the years 1998, 2003 and 2008 were measured using the question: “In the past 12 months, have you seen a doctor?” (yes/no). In 2013, the question used was: “When did you last see a doctor?” and the options were: (i) in the last 12 months, (ii) from 1 year to less than 2 years, (iii) from 2 years to less than 3 years, (iv) 3 years or more, and (v) never went to the doctor. To standardize the responses, the 2013 survey variable was recategorized as ‘yes’ for those who answered affirmatively for option (i) and ‘no’ for those who opted for the other responses (ii, iii, iv and v).

Multimorbidity was assessed by counting the number of self-reported morbidities, following the definition most used in the literature, that is, the presence of two or more chronic problems in the same individual [22, 23]. The following morbidities were included in the list: (a) chronic back problem, (b) arthritis or rheumatism, (c) cancer, (d) diabetes, (e) bronchitis or asthma, (f) arterial hypertension, (g) heart disease, (h) chronic kidney disease, (i) depression and (j) tendinitis/tenosynovitis. All morbidities weighted 1.0 (one) in the total morbidity count. From the count, multimorbidity was recognised as the presence of two or more morbidities (yes) and one or no morbidity (no).

The selection of independent variables was based on the behavioral model for the use of health services by Andersen (1995) [24], which classifies the variables into predisposing, enabling factors and health needs. Predisposing factors were assessed using the variables gender (female/male), categorized age (18–29, 30–39, 40–49, 50–59, 60–69 and ≥ 70 years), education (no schooling, incomplete elementary school, complete elementary school, incomplete high school, complete high school, incomplete university and completed university), and race/color (white, brown, black, yellow and indigenous).

The enabling factors were measured through possession of a health plan (yes/no) and registration primary care in family health teams (FHT) (yes/no), for the latter there is information only for the years, 2008 and 2013. Health needs were assessed through self-assessment of health status (very good, good, fair, poor and very bad) and limitation of usual activities in the last 15 days (yes/no). The questions regarding the study variables according to the surveys are available in the supplementary material (Additional file 1).

### Data analysis

Prevalence was estimated for each outcome of health service use stratified by multimorbidity according to the independent variables, for each year of the panel. The Wald test was used for the linear trend between the outcome and the independent variables. Trends with a *p*-value of less than 0.05 were considered statistically significant. Poisson regression models stratified by sex were used to estimate the crude and adjusted prevalence ratios (PR) and their respective 95% confidence intervals (95% CI) for each outcome of health service use and multimorbidity, per year. The initial models were progressively adjusted for predisposing, enabling factors, and health needs. The Stata SE 15.0 application (College Station, TX, USA) was used for statistical analysis, and the sample parameters and weights of individuals were considered in all analyses.

## Results

The proportion of women was higher in all years of study and the largest population group was aged between 18 and 29 years, with a greater number of individuals aged 60 years or older in 2013. In all years, the majority of the population declared themselves as belonging to white color or race, but in 2013, blacks and browns together represented more than half of the population studied (Table 1). The relative frequency of individuals with low education has decreased over the years and there has been an increase in the proportion of people with university education. The possession of a health plan and registration with FHT increased over the study period, reaching approximately 30 and 60% of the population respectively, in 2013(Table 1).

**Table 1:** Demographic, socioeconomic, multimorbidity, and health services characteristics of the study population by year. Brazil, 1998–2013

Variables	1998	2003	2008	2013
	*N* = 100,222,274	*N* = 118,463,739	*N* = 134,105,731	*N* = 124,010,200
	% (95%CI)	% (95% CI)	% (95% CI)	% (95% CI)
**Sex**				
Male	47.9 (47.7–48.2)	47.7 (47.5–47.9)	47.7 (47.5–47.9)	47.1 (46.7–47.5)
Female	52.1 (51.8–52.3)	52.3 (52.1–52.5)	52.3 (52.1–52.5)	52.9 (52.5–53.3)
**Age**				
18–29	32.5 (32.3–32.7)	32.3 (32.1–32.5)	29.7 (29.5–29.9)	26.5 (26.1–26.9)
30–39	23.4 (23.2–23.6)	22.2 (22.0–22.3)	21.2 (21.0–21.3)	21.1 (20.8–21.5)
40–49	18.3 (18.1–18.5)	18.6 (18.4–18.7)	19.1 (19.0–19.3)	18.6 (18.2–18.9)
50–59	12.0 (11.8–12.1)	12.6 (12.4–12.7)	14.1 (14.0–14.3)	15.7 (15.4–16.0)
60–69	8.0 (7.8–8.1)	8.0 (7.9–8.1)	8.8 (8.7–8.9)	10.1 (10.0–10.4)
> =70	5.9 (5.8–6.0)	6.4 (6.3–6.5)	7.1 (7.0–7.2)	8.0 (7.7–8.2)
**Race/skin color**				
White	56.4 (56.1–56.6)	53.9 (53.7–54.1)	50.0 (49.8–50.2)	47.6 (47.2–47.9)
Brown	36.6 (36.4–36.8)	39.0 (38.8–39.2)	41.5 (41.3–41.7)	42.0 (41.6–42.4)
Black	6.2 (6.1–6.3)	6.4 (6.3–6.5)	7.6 (7.4–7.7)	9.2 (8.9–9.4)
Yellow	0.7 (0.6–0.7)	0.5 (0.5–0.6)	0.7 (0.6–0.7)	0.9 (0.8–0.9)
Indigenous	0.2 (0.2–0.2)	0.2 (0.2–0.2)	0.3 (0.3–0.3)	0.4 (0.4–0.5)
**Education**				
No schooling	5.2 (5.1–5.3)	5.1 (5.0–5.2)	5.4 (5.3–5.5)	13.7 (13.5–14.0)
Incomplete elementary school	52.2 (52.0–52.5)	44.3 (44.1–44.5)	36.2 (36.0–36.5)	25.6 (25.3–26.0)
Complete elementary school	8.9 (8.7–9.0)	8.7 (8.5–8.8)	9.1 (9.0–9.2)	9.8 (9.6–10.1)
Incomplete high school	7.8 (7.7–7.9)	8.5 (8.4–8.6)	8.1 (8.0–8.2)	5.4 (5.3–5.6)
Complete high school	15.6 (15.4–15.8)	20.3 (20.1–20.5)	24.9 (24.7–25.1)	27.5 (27.1–27.9)
Incomplete university	3.8 (3.7–3.9)	5.5 (5.4–5.6)	6.6 (6.5–6.7)	5.2 (5.0–5.4)
Completed university	6.6 (6.5–6.7)	7.6 (7.5–7.8)	9.8 (9.7–10.0)	12.1 (12.4–13.0)
**Health insurance**				
Yes	26.6 (26.4–26.8)	26.9 (26.7–27.1)	28.1 (27.9–28.3)	29.9 (29.5–30.2)
No	73.4 (73.2–73.6)	73.1 72.9–73.3)	71.9 (71.7–72.1)	70.1 (69.8–70.5)
**Registered in FHT**				
Yes	–	–	48.8 (48.6–48.9)	61.1 (60.7–61.4)
No	–	–	51.3 (51.1–51.4)	38.9 (38.6–39.3)
**Health self-assessment**				
Very good	22.3 (22.1–22.5)	20.6 (20.4–20.7)	18.5 (18.3–18.7)	12.6 (12.3–12.9)
Good	49.3 (49.1–49.6)	51.8 (51.6–52.0)	52.8 (52.6–53.0)	56.1 (55.7–56.5)
Regular	23.1 (22.9–23.3)	22.9 (22.7–23.1)	23.6 (23.4–23.7)	25.7 (25.4–26.1)
Poor	4.4 (4.3–4.5)	3.9 (3.8–4.0)	4.1 (4.0–4.2)	4.6 (4.4–4.7)
Very poor	0.9 (0.8–0.9)	0.8 (0.8–0.8)	1.0 (1.0–1.1)	1.0 (0.9–1.1)
**Limitation of usual activities**				
Yes	7.2 (7.1–7.3)	7.3 (7.2–7.4)	8.9 (8.8–9.0)	7.5 (7.3–7.8)
No	92.8 (92.7–92.9)	92.7 (92.6–92.8)	91.1 (91.0–91.2)	92.5 (92.2–92.7)
**Multimorbidity**				
Yes	21.5 (21.3–21.7)	18.1 (18.0–18.3)	18.5 (18.3–18.6)	22.1 (21.6–22.7)
No	78.5 (78.3–78.7)	81.9 (81.7–82.0)	81.5 (81.4–81.7)	77.9 (77.3–78.4)
**Search for health services**				
Yes	13.2 (13.0–13.3)	14.8 (14.7–15.0)	14.1 (13.9–14.2)	19.8 (19.3–20.4)
No	86.8 (86.7–87.0)	85.2 (85.0–85.3)	85.9 (85.8–86.1)	80.2 (79.6–80.7)
**Medical appointment**				
Yes	57.6 (57.3–57.8)	64.6 (64.4–64.8)	69.8 (69.7–70.0)	78.6 (78.0–79.1)
No	42.4 (42.2–42.7)	35.4 (35.2–35.6)	30.2 (30.0–30.4)	21.4 (20.9–22.0)
**Hospitalisations**				
Yes	8.4 (8.3–8.6)	8.1 (7.9–8.2)	8.0 (7.9–8.1)	7.9 (7.6–8.3)
No	91.6 (91.4–91.7)	91.9 (91.8–92.1)	92.0 (91.9–92.1)	92.1 (91.7–92.4)

Over the years, there has been an increase in the proportion of people who rated their health as good, reaching 56% in 2013, and the proportion of people who reported limitation in their usual activities was on an average 8% during the study period. Approximately 20% of the population was classified as having multimorbidity during the study period. The demand for health services increased from 13% in 1998 to 17% in 2013. Medical consultations increased by 14% between 1998 and 2013. Finally, the percentage of hospitalisations decreased over the years, reaching 7% in 2013 (Table 1).

Table 2 shows the prevalence of searching health services in the last 15 days according to the multimorbidity classification. Among individuals with multimorbidity, the prevalence of seeking services was higher among women, older age groups, whites, and browns, those with the lowest education levels, those who had health insurance and registration in primary care FHT. The prevalence of searching services was also higher among individuals who rated their health as very poor and presented limitations in their usual activities in the last 15 days. Also, an increase in the prevalence of searching for services over the years for all variables could be noticed, regardless of the multimorbidity classification. However, the prevalence of searching health services among individuals with multimorbidity was almost double that of individuals without multimorbidity, except for those who reported limitations on their usual activities (Table 2).

**Table 2:** Prevalence of searching health services in the last 15 days stratified by multimorbidity. Brazil, 1998–2013

Variables	Multimorbidity					Without multimorbidity				
	1998	2003	2008	2013		1998	2003	2008	2013	
	%	%	%	%	*p*-value^a^	%	%	%	%	*p*-value^a^
	25.95	31.54	29.95	34.44	***	9.64	11.15	10.5	15.65	***
**Sex**										
Male	20.57	27.17	26.8	29.88	***	6.67	7.77	8.01	11.86	***
Female	29.05	33.89	31.71	36.48	***	12.73	14.59	13.01	18.58	***
**Age**										
18–29	22.49	28.4	28.77	33.7	***	8.82	9.68	9.27	13.25	***
30–39	23.87	28.31	27.82	34.13	***	9.34	10.9	10.04	14.06	***
40–49	25.6	30.84	30	34.26	***	9.94	11.91	10.76	15.58	***
50–59	25.84	31.86	30.73	35.41	***	10.69	12.4	11.96	16.31	***
60–69	27.78	32.68	29.35	32.82	***	11.81	14.18	12.54	20.77	***
> =70	28.07	33.9	30.89	35.57	***	13.93	16.14	15.33	22.23	***
**Race/skin color**										
White	27.44	32.35	29.93	35.54	***	10.07	11.59	10.93	16.01	***
Brown	25.26	31.88	30.77	36.33	***	9.15	11.35	10.43	14.4	***
Black	25.58	26.84	22.56	41.03		7.63	10.51	11.04	19.03	**
Yellow	23.84	30.28	30.05	31.9	***	9.08	10.53	9.98	15.44	***
Indigenous	35.37	28.09	25.95	56.17		10.85	12.68	13.93	12.06	
**Education**										
No schooling	24.21	33.36	31.38	33.95	***	8.55	11.27	10.89	16.34	***
Incomplete elementary school	26.39	31.54	30.19	33.42	***	9.02	10.91	10.51	16.59	***
Complete elementary school	28.02	30.63	30.42	40.36	***	9.44	10.2	10.01	13.43	***
Incomplete high school	26.33	29.85	29.3	27.52		9.48	9.65	9.42	13.96	**
Complete high school	27.72	30.53	29.53	34.7	**	10.56	11.37	10.09	14.6	***
Incomplete university	24.45	29.27	27.92	35.07		11.33	12.02	10	15.07	***
Completed university	31.04	32.38	28.67	34.65	**	12.72	13.9	12.39	17.93	***
**Health insurance**										
Yes	32.4	35.14	32.32	36.89	***	14.0	15.1	13.3	18.85	***
No	23.89	30.03	28.93	33.18	***	8.0	9.74	9.42	14.11	***
**Registered in FHT**										
Yes	–	–	30.96	34.12		–	–	10.87	16.23	
No	–	–	28.91	34.16		–	–	10.15	15.49	
**Health self-assessment**										
Very good	14.92	19.32	18.25	23.79	***	6.24	7.33	6.93	12.66	***
Good	16.34	21.83	21.16	27.19	***	8.4	9.52	8.78	13.25	***
Regular	26.79	32.82	30.66	36.31	***	18.0	19.65	18.33	22.35	***
Poor	39.1	46.95	41.64	45.05	***	26.54	29.56	28.51	26.14	*
Very poor	47.7	56.48	51.1	49.27	***	29.27	29.43	34.15	35.99	
**Limitation of usual activities**										
Yes	58.05	65	60.24	62.37	***	56.08	56.87	52.23	63.36	***
No	18.8	23.63	21.37	27.8	***	7.62	8.89	7.86	12.39	***

Note: ^a^: Test Wald (Pearson) adjusted for the linear tendency; *p*-value: * ≤ 0.05, ** ≤ 0.01, *** ≤ 0.001

The prevalence of medical consultations in the last 12 months between 1998 and 2013, showed an increasing trend over the years for all variables analysed. The prevalence of medical appointments did not differ much with regard to sex, education, having health insurance, and limiting usual activities in the last 15 days among individuals who were classified as having multimorbidity. However, when comparing the prevalence of use for these same variables among individuals without multimorbidity, higher prevalence was observed among women, individuals with higher levels of education, individuals having a health plan, and individuals who reported limitation in the usual activities in the last 15 days (Table 3).

**Table 3:** Prevalence of medical appointments in the last 12 months stratified by multimorbidity. Brazil, 1998–2013

Variables	Multimorbidity					Without multimorbidity				
	1998	2003	2008	2013		1998	2003	2008	2013	
	%	%	%	%	p-value^a^	%	%	%	%	p-value^a^
	79.47	87.62	89.94	92.36	***	51.56	59.47	65.29	74.64	***
Mean annual of consultations	5.8	6.6	6.7	6.0		3.5	3.6	3.7	3.6	
**Sex**										
Male	71.38	82.19	85.48	88.23	***	40.82	47.83	53.66	67.2	***
Female	84.14	90.53	92.44	94.21	***	62.75	71.38	77.08	80.39	***
**Age**										
18–29	75.76	83.8	86.54	84.52	***	47.65	55.11	60.99	73.04	***
30–39	77.01	84.98	87.7	90.47	***	52.13	60.08	65.99	73.56	***
40–49	77.86	86.51	88.9	90.69	***	53.33	62.05	67.19	73.13	***
50–59	79.63	87.61	89.96	92.13	***	55.39	62.95	68.24	75.25	***
60–69	81.97	89.3	90.82	94.01	***	59.24	65.1	69.94	79.1	***
> =70	82.54	90.1	91.83	94.63	***	61.94	69.63	74.29	81.68	***
**Race/skin color**										
White	81.78	88.47	90.85	93.39	***	54.1	61.96	67.46	76.45	***
Brown	79.57	87.79	89.96	91.27	***	47	57.68	64.58	72.65	***
Black	84.75	87.49	88.18	91.17		50.91	61.47	65.88	73.56	***
Yellow	75.99	86.24	88.74	91.01	***	48.37	56.39	62.93	72.9	***
Indigenous	79.38	84.57	87.7	96.51	**	50.17	61.65	64.36	81.01	***
**Education**										
No schooling	73.59	86.01	88.22	92.07	***	45.06	51.27	57.53	70.57	***
Incomplete elementary school	80.35	87.55	90.2	92.03	***	48.36	56.5	62.32	71.82	***
Complete elementary school	82.95	88.3	89.35	94.32	***	50.48	57.91	63.4	72.27	***
Incomplete high school	80.38	87.61	91.02	89.97	***	50.92	56.61	61.04	72.04	***
Complete high school	83.44	88.08	90.83	91.02	***	57.98	63.07	67.34	75.06	***
Incomplete university	84.09	88.85	89.17	92.17	*	61.45	67.99	70.04	76.28	***
Completed university	88.84	91.96	93.14	94.86	***	67.63	75.17	78.5	83.1	***
**Health insurance**										
Yes	89.27	93.07	94.31	96.59	***	69	75.79	78.45	86.44	***
No	76.35	85.32	88.07	90.18	***	45.01	53.66	60.24	68.97	***
**Registered in FHT**										
Yes	–	–	90.04	92.15		–	–	64.56	75.19	
No	–	–	89.84	92.74		–	–	66	74.81	
**Health self-assessment**										
Very good	68.25	81.56	85	89.48	***	43.35	53.36	59.17	71.97	***
Good	71.92	83.24	87.43	89.61	***	50.4	57.53	63.79	72.86	***
Regular	81.42	89.04	90.44	93.48	***	67.6	72.28	75.9	79.97	***
Poor	87.38	92.52	93.08	94.68	***	74.84	78.19	82.05	83.24	***
Very poor	89.83	94.67	94.3	97.18	***	70.38	77.39	82.36	82.56	***
**Limitation of usual activities**										
Yes	90.93	95.39	95.51	95.46	***	84	86.55	86.77	93.47	***
No	76.92	85.78	88.36	91.62	***	50.15	58.13	63.93	73.35	***

Note: ^a^: Test Wald (Pearson) adjusted for the linear tendency; *p*-value: * ≤ 0.05, ** ≤ 0.01, *** ≤ 0.001

The prevalence of hospitalisations decreased over the study period, however, the prevalence of hospitalisation among those individuals characterised with multimorbidity was double that of the individuals without multimorbidity. The prevalence of hospitalisations among men with multimorbidity was higher compared to women in 2003 and almost triple compared to men without multimorbidity in 2013. The highest prevalence of hospitalisations among individuals with multimorbidity was at the lowest educational levels. Finally, the differences in the prevalence of hospitalisations according to possession of a health plan decreased over the years among individuals who had multimorbidity, reaching less than 1% in the last year of study (Table 4).

**Table 4:** Prevalence of hospitalisations in the last 12 months stratified by multimorbidity. Brazil, 1998–2013

Variables	Multimorbidity					Without multimorbidity				
	1998	2003	2008	2013		1998	2003	2008	2013	
	%	%	%	%	p-value^a^	%	%	%	%	p-value^a^
	15.03	15.74	15.68	13.44	***	6.62	6.34	6.29	6.38	***
Mean annual of hospitalisations	1.6	1.7	1.7	1.7		1.2	1.3	1.3	1.3	
**Sex**										
Male	14.44	16.21	16.21	13.87	***	3.95	4.24	4.50	4.78	***
Female	15.37	15.49	15.39	13.24	**	9.41	8.50	8.11	7.63	***
**Age**										
18–29	17.41	17.57	17.78	15.8		7.57	6.79	6.93	7.05	***
30–39	13.02	14.02	14.82	11.7	*	6.27	6.41	6.24	6.44	
40–49	12.18	13.6	14.25	11.1	***	4.78	5.19	5.07	5.23	
50–59	13.62	14.38	14.27	11.14	**	5.37	5.19	5.30	5.92	
60–69	15.67	15.43	14.81	14.36		7.53	6.34	5.96	6.07	***
> =70	20.46	20.42	19.54	17.17		10.29	10.20	10.19	8.67	
**Race/skin color**										
White	15.34	15.67	15.66	13.00	**	6.63	6.20	6.23	6.27	***
Brown	13.62	15.68	15.35	13.78		5.86	6.13	5.88	5.65	
Black	17.74	12.15	11.5	13.18		4.44	6.02	6.06	7.84	
Yellow	14.8	15.9	15.86	14.02	**	6.76	6.57	6.44	6.67	
Indigenous	16.46	17.27	20.38	14.29		7.97	7.53	8.16	4.60	
**Education**										
No schooling	15.68	18.14	18.7	17.36	**	7.66	7.69	6.88	8.31	
Incomplete elementary school	15.08	15.35	15.08	13.04		6.96	6.50	6.62	5.95	**
Complete elementary school	13.33	12.95	15.6	12.59	*	6.55	6.02	6.58	6.62	
Incomplete high school	13.46	15.00	14.66	11.91		5.23	5.89	6.17	7.96	***
Complete high school	12.92	14.14	13.86	11.06		6.43	6.13	5.82	6.02	*
Incomplete university	13.85	13.22	15.35	11.34		5.04	4.81	5.04	5.29	
Completed university	13.88	15.15	14.76	12.81		5.87	6.14	6.36	5.95	
**Health insurance**										
Yes	16.96	17.18	17.05	12.84	***	7.56	7.20	7.08	7.38	
No	14.42	15.13	15.1	13.74	**	6.27	6.04	5.99	5.90	*
**Registered in FHT**										
Yes	–	–	16.68	14.17		–	–	6.86	6.76	
No	–	–	14.65	12.43		–	–	5.75	5.87	
**Health self-assessment**										
Very good	7.66	8.91	9.35	11.00		4.71	4.50	4.34	5.10	*
Good	8.84	9.61	9.99	9.99	*	5.89	5.57	5.47	5.60	**
Regular	14.58	15.52	15.10	12.57	***	10.85	9.85	9.81	8.33	***
Poor	26.20	27.54	25.33	20.52	***	20.67	19.00	18.97	12.52	***
Very poor	33.37	37.98	34.44	32.52		25.32	22.03	23.21	17.53	
**Limitation of usual activities**										
Yes	29.12	31.47	28.64	25.52	***	22.94	21.91	20.08	19.48	***
No	11.89	12.02	12.01	10.56		5.913	5.574	5.42	5.49	***

Note: ^a^: Test Wald (Pearson) adjusted for the linear tendency; *p*-value: * ≤ 0.05, ** ≤ 0.01, *** ≤ 0.001

The crudes and adjusted PRs for the use of health services (search for services, medical consultations, and hospitalisations) for each year of study stratified by sex are shown in Table 5. It is possible to verify that having multimorbidity was associated with a threefold increase in the probability of searching health care among men and a twofold increase for women in 1998.

**Table 5:** Crude and adjusted prevalence ratios for the use of health services. Brazil, 1998–2013

Variable	Male				Female			
	Model 1	Model 2	Model 3	Model 4	Model 1	Model 2	Model 3	Model 4
	PR (95% CI)	PR (95% CI)	PR (95% CI)	PR (95% CI)	PR (95% CI)	PR (95% CI)	PR (95% CI)	PR (95% CI)
**Search for health services**								
1998	3.08	2.66	2.57	1.38	2.28	2.21	2.24	1.47
	(2.95–3.22)	(2.52–2.81)	(2.44–2.69)	(1.31–1.45)	(2.22–2.35)	(2.14–2.29)	(2.17–2.31)	(1.42–1.52)
2003	3.49	2.75	2.73	1.58	2.32	2.16	2.18	1.47
	(3.37–3.62)	(2.63–2.89)	(2.61–2.85)	(1.51–1.65)	(2.27–2.38)	(2.09–2.22)	(2.12–2.24)	(1.42–1.51)
2008	3.34	2.67	2.66	1.51	2.44	2.35	2.34	1.52
	(3.23–3.46)	(2.55–2.80)	(2.56–2.78)	(1.45–1.58)	(2.38–2.50)	(2.28–2.43)	(2.27–2.41)	(1.48–1.57)
2013	2.52	2.09	1.98	1.46	1.96	1.85	1.81	1.39
	(2.26–2.81)	(1.85–2.36)	(1.75–2.25)	(1.28–1.67)	(1.84–2.10)	(1.72–1.99)	(1.68–1.96)	(1.29–1.51)
**Doctor’s appointments**								
1998	1.75	1.59	1.59	1.28	1.34	1.32	1.33	1.19
	(1.72–1.77)	(1.56–1.61)	(1.56–1.61)	(1.26–1.30)	(1.33–1.35)	(1.31–1.34)	(1.31–1.34)	(1.18–1.20)
2003	1.72	1.52	1.50	1.28	1.27	1.23	1.22	1.14
	(1.70–1.74)	(1.50–1.54)	(1.48–1.52)	(1.26–1.30)	(1.26–1.28)	(1.22–1.24)	(1.22–1.23)	(1.14–1.15)
2008	1.59	1.45	1.44	1.24	1.20	1.17	1.17	1.11
	(1.58–1.61)	(1.43–1.47)	(1.42–1.45)	(1.22–1.25)	(1.19–1.21)	(1.16–1.18)	(1.16–1.17)	(1.10–1.11)
2013	1.31	1.25	1.23	1.16	1.17	1.16	1.15	1.11
	(1.28–1.35)	(1.21–1.29)	(1.19–1.26)	(1.12–1.20)	(1.16–1.19)	(1.14–1.18)	(1.13–1.17)	(1.09–1.12)
**Hospitalisations**								
1998	3.66	2.67	2.66	1.43	1.63	1.79	1.80	1.30
	(3.47–3.86)	(2.48–2.86)	(2.48–2.86)	(1.32–1.55)	(1.57–1.70)	(1.71–1.88)	(1.72–1.88)	(1.23–1.36)
2003	3.83	2.79	2.76	1.63	1.82	2.05	2.02	1.44
	(3.64–4.02)	(2.61–2.98)	(2.59–2.95)	(1.52–1.75)	(1.76–1.89)	(1.96–2.14)	(1.93–2.11)	(1.37–1.51)
2008	3.60	2.76	2.72	1.54	1.90	2.16	2.13	1.52
	(3.43–3.78)	(2.59–2.94)	(2.55–2.90)	(1.44–1.64)	(1.83–1.97)	(2.06–2.25)	(2.04–2.23)	(1.45–1.60)
2013	2.90	2.19	2.18	1.55	1.74	1.85	1.83	1.45
	(2.45–3.44)	(1.83–2.63)	(1.80–2.65)	(1.26–1.90)	(1.54–1.95)	(1.64–2.10)	(1.60–2.09)	(1.25–1.68)

Note: Model 1: crude analysis; Model 2: adjusted for age, education and race/color; Model 3: Model 2 + possession of a health insurance and FHT registration; Model 4: Model 3 + self-assessment of health status and limitation of usual activities

Behind progressive adjustment due to predisposing factors, enabling factors and health needs, a reduction in the strength of association between the search for services and multimorbidity was observed over the years, regardless of gender. However, for men having multimorbidity, the search for services increased by 38% in 1998 and 46% in 2013. On the contrary, among women, having multimorbidity increased the search by 47% in 1998 and decreased to 39% in 2013.

Having multimorbidity increased the chance of having medical appointments in the past 12 months, but the association has decreased over the years, regardless of gender. After adjustment, for men, having multimorbidity increased the chance of medical consultations in 1998 by 28%, reducing to 16% in 2013. Among women, this association was 19% in 1998 and 11% in 2013 (Table 5).

Regarding hospitalisations, in the bivariate analysis, the chance of being hospitalised among men who had multimorbidity was approximately 4 times greater compared to those who did not have multimorbidity in 1998. After progressive adjustment, there was a reduction in the association between having multimorbidity and having been hospitalised, regardless of gender. Among men, having multimorbidity increased the chance of having been hospitalised by 63% in 2003, decreasing to 55% in 2013. In women, this association was 44% higher among those with multimorbidity in 2003, and 45% in 2013 (Table 5).

## Discussion

There was an increase in the prevalence of searching health services and medical appointments in the last 12 months between 1998 and 2013, regardless of the multimorbidity classification. On the other hand, there was a reduction in the prevalence of hospitalisations during the study period. Despite the similar trend of growth in the prevalence of searching health services, among individuals with multimorbidity, there is twice as high prevalence to those without multimorbidity for all the conditions studied, except for those who reported limitation in their usual activities in the last 15 days. However, for medical consultations, the prevalence rates did not differ with regard to sex, education, having a health plan and limiting usual activities in the last 15 days among individuals with multimorbidity.

Besides, it is noteworthy that despite the reduction in hospitalisations over the years, the prevalence of hospitalisations among men with multimorbidity was higher compared to women from 2003 and almost threefold compared to men without multimorbidity in 2013. Having multimorbidity increased the search for health services by 46% for men and 39% for women in the last year of study. This relationship increased the chances for medical appointments by 16% for men and 11% for women in 2013. Finally, having multimorbidity increased the chance of being hospitalised by 55% for men and 45% for women in the year 2013.

In Brazil, NCD carriers use health services more [25]. Access to and use of health services depend on a set of factors that can be divided into determinants of supply and demand [26]. The perceived need, that is, the identification of a problem by the user, is the most important driver of demand and usually overlaps other demographic and social characteristics [26, 27]. In the case of multimorbidity, our study showed that the prevalence of searching for services in the last 15 days was twice as high as those without multimorbidity, regardless of the sociodemographic characteristic analysed.

Also, the high prevalence of searching health services in this population can be explained in part, by the components of the provision of health services. In Brazil and most parts of the world, health systems are designed around unique conditions or body systems [28]. This focus extends to the training of health professionals, particularly those who work in hospitals where specialization is common, leaving the coordination of care for patients with multiple chronic conditions to family doctors, general practitioners and geriatricians [7, 28]. This health care model can motivate a greater number of visits to different services by the same individual, overloading the health system.

Regarding medical consultations in the last 12 months, according to Viacava and Bellido (2016) [29] in 2013, 71.2% of the Brazilian population reported having had a medical consultation in the last 12 months; and in all regions of the country, except for the North, the increase in the prevalence of medical consultations was significant, between 1998 and 2013. In the general population, the use of health services is higher among adults with private insurance, among women and for people with a higher level of education in all years [30]. However, similar to our study, a study carried out in Serbia [31] found that having multimorbidity reduced the differences in the prevalence of medical visits in these variables, indicating a possible reduction in inequalities in the use of health services in populations with greater health needs, such as the case of people with multimorbidity.

The use of secondary services, measured as utilizing hospitalisations, had a prevalence twice as high among individuals classified as having multimorbidity and had a different pattern with regard to sex. In general, our results are in line with the findings of other studies, which point to a twice as high probability of hospitalisations among individuals with multimorbidity [13, 31–33].

Among the three health service utilization outcomes measured by this study, only the prevalence of hospitalisations was higher among men than women. According to Hulka and Wheat (1985) [34], the use of health services can be explained mainly by the profile of the health needs of a population group. It is already known that in the general population, women make more visits to primary care centres than men and seek more services for routine exams and prevention, while men seek health services predominantly due to illness [35, 36].

The study by Jankovic et al. (2018) [31], found a three times greater probability of having a medical consultation (OR = 3.17 in men, OR = 3.14 in women) and two times greater probability of having been hospitalised in the last 12 months (OR = 2.45 in men, OR = 1.97 in women) in the Serbian population, among individuals with multimorbidity compared to those without any condition.

In our study, even after progressive adjustment of predisposing factors, enabling factors and health needs, having multimorbidity increased the chance of using health services for the three outcomes analysed, with greater influence among men. Our findings corroborate the results of other studies [12, 13, 15, 32, 37, 38], showing an increase in the use of health services in primary and secondary care associated with multimorbidity, even when controlling for age, sex and social status.

The study’s limitations include the use of self-reported clinical conditions for chronic diseases and the use of health services that may underestimate their prevalence [8, 39]. Furthermore, in defining multimorbidity as a simple count of NCDs, our study considered all diseases equally, although the effect of multimorbidity on individuals may vary with the combination and severity of NCDs [7]. Additionally, it should be noted that the list of self-reported morbidities used for the classification of simple count addressed only 10 diagnoses, a fact that may have reduced the estimates of multimorbidity among the individuals evaluated.

This study represents one of the first detailed descriptions of the effect of multimorbidity on the use of health services in Brazil. Among the strengths, this study included data of national scope that make it possible to generalise our results to the entire population and even to other countries with similar characteristics. Also, the analyses including four points in time made it possible to infer trends in the use of services, and the very similar issues in the 15 years analysed allowed to maintain comparability.

## Conclusion

Multimorbidity is increasingly becoming common worldwide, with increasing implications for the management of patients, assessment of disease burden in populations and the efficiency and effectiveness of health systems [7, 40]. This study found that individuals with multimorbidity have higher levels of use of health services and, in the process, can often be seen by several health professionals.

To increase the quality of care provided to these patients and reduce the rising costs of health care, it is necessary to focus on continuous, coordinated and comprehensive approaches to the care of people with multimorbidity through the health system. This requires a change from current approaches, which often emphasize specific vertical disease programs.

Primary care services are the ideal setting for this process to occur, given their fundamental role in providing continuous, well-coordinated and comprehensive care to patients with complex health needs, including those with multiple NCDs.

Finally, more research is needed to better understand the epidemiology of multimorbidity and the associated impacts on the use and costs of health services in Brazil. Evidence on the patterns of use of health services contributes to the improvement of the health system, in the improvement of the management of individuals with multimorbidity aiming at better health results, thus increasing the efficiency of the assistance provided and reducing costs.

## Supplementary Information

**Additional file 1.**

## Data Availability

The PNAD and PNS microdata are accessible to the public and available on the IBGE website. • https://www.ibge.gov.br/estatisticas/sociais/saude/19898-suplementos-pnad3.html?=&t=microdados • https://www.ibge.gov.br/estatisticas/sociais/saude/9160-pesquisa-nacional-de-saude.html?=&t=microdados

## Abbreviations

NCDs: Chronic non-communicable diseases
PNAD: Brazilian National Household Sample Survey
PNS: Brazilian National Health Survey
IBGE: Brazilian Institute Geography and Statistics
FHT: Family health teams
PR: Prevalence ratios
95%CI: 95% confidence intervals
OR: Odds ratio
