# Supplementary material for: Trends in the use of health services and their relationship with multimorbidity in Brazil, 1998–2013
Source: BMC Health Serv Res. 2020 Nov 25;20:1080. doi: 10.1186/s12913-020-05938-4 (PMC7690184; doi:10.1186/s12913-020-05938-4)
Supplement: Supplementary file 1 — Additional file 1. [file 12913_2020_5938_MOESM1_ESM.docx]

Study variables and their respective questions according to the surveys - PNAD 1998, 2003, 2008, and PNS 2013.

| VARIABLES | PNAD 1998 | PNAD 2003 | PNAD 2008 | PNS 2013 |
| --- | --- | --- | --- | --- |
| Chronic back problem | V1309 - Do you have spine or back disease? | V1309 - Did any doctor or health professional say you have spine or back disease? | V1309 - Did any doctor or health professional say you have spine or back disease? | Q084 - Do you have any chronic back problems, such as chronic back or neck pain, low back pain, sciatica, vertebrae, or disc problems? |
| Arthritis or rheumatism | V1310 - Do you have arthritis or rheumatism? | V1310 - Did any doctor or health professional say you have arthritis or rheumatism? | V1310 - Did any doctor or health professional say you have arthritis or rheumatism? | Q079 - Has any doctor ever given you a diagnosis of arthritis or rheumatism? |
| Cancer | V1311 - Do you have cancer? | V1311 - Did any doctor or health professional say you have cancer? | V1311 - Did any doctor or health professional say you have cancer? | Q120 - Has any doctor ever given you a cancer diagnosis? |
| Diabetes | V1312 - Do you have diabetes? | V1312 - Did any doctor or healthcare professional say you have diabetes? | V1312 - Did any doctor or healthcare professional say you have diabetes? | Q030 - Has a doctor ever diagnosed you with diabetes? |
| Bronchitis or asthma | V1313 - Do you have bronchitis or asthma? | V1313- Did any doctor or healthcare professional say you have bronchitis or asthma? | V1313 - Did any doctor or healthcare professional say you have bronchitis or asthma? | Q074 - Has any doctor ever given you a diagnosis of asthma (or asthmatic bronchitis)? |
| Arterial hypertension | V1314 - Do you have hypertension (high blood pressure)? | V1314 - Did any doctor or health professional say you have hypertension? | V1314 - Did any doctor or health professional say you have hypertension? | Q002 - Has any doctor ever given you a diagnosis of high blood pressure (high blood pressure)? |
| Heart disease | V1315 - Do you have heart disease? | V1315 - Did any doctor or health professional say you have heart disease? | V1315 - Did any doctor or health professional say you have heart disease? | Q063 - Has any doctor ever diagnosed you with heart disease such as a heart attack, angina, heart failure, or other? |
| Chronic kidney disease | V1316 - Do you have chronic kidney disease? | V1316 - Did any doctor or health professional say you have chronic kidney failure? | V1316 - Did any doctor or health professional say you have chronic kidney failure? | Q124 - Has any doctor ever given you a diagnosis of chronic kidney failure? |
| Depression | V1317 - Do you have depression? | V1317 - Did any doctor or health professional say you have depression? | V1317 - Did any doctor or health professional say you have depression? | Q092 - Has a doctor or mental health professional (such as a psychiatrist or psychologist) ever diagnosed you with depression? |
| Tendinitis/tenosynovitis | V1319 - Do you have tendonitis or tenosynovitis? | V1319 - Did any doctor or healthcare professional say you have tendonitis or tenosynovitis? | V1319 - Did any doctor or healthcare professional say you have tendonitis or tenosynovitis? | Q088 - Has any doctor ever given you a diagnosis of WMSD (work-related musculoskeletal disorder)? |
| Search for health services in the last 15 days | V1350 - In the past two weeks, have you looked for a place, service, or health professional for care related to your own health? | V1350 - In the past two weeks, have you looked for a place, service, or health professional for care related to your own health? | V1350 - In the past two weeks, have you looked for a place, service, or health professional for care related to your own health? | J014 - In the past two weeks, did _____ seek any place, service, or health professional for care related to their own health? |
| Medical consultation in the last 12 months | V1347 - Have you seen a doctor in the last 12 months? | V1347 - Have you seen a doctor in the last 12 months? | V1347 - Have you seen a doctor in the last 12 months? | J011 - When did ________ last see a doctor? |
| Hospitalisations in the last 12 months | V1369 - In the last 12 months, have you been hospitalised? | V1369 - In the last 12 months, have you been hospitalised? | V1369 - In the last 12 months, have you been hospitalised? | J037 - In the past 12 months, did _______ stay in the hospital for 24 hours or more? |
| Gender | V0302 – Gender | V0302 - Gender | V0302 - Gender | C006 - Gender |
| Age | V8005 - Resident's age | V8005 - Resident's age | V8005 - Resident's age on the reference date | C008 - Age |
| Race/color | V0404 – Color or race | V0404 – Color or race | V0404 – Color or race | C009 – Color or race |
| Education | V 4702 - Grade and Series they attended - II | V4702 - Grade and Series they attended - II | V4802 - Grade and Series they attended - II | VDD004 - Highest level of education reached (people aged 5 and over) |
| Possession of a health plan | V1321 - Are you entitled to any health plan (medical or dental), private, company, or public agency? | V1321 - Are you entitled to any health plan (medical or dental), private, company, or public agency? | V1321 - Are you entitled to any health plan (medical or dental), private, company, or public agency? | I001 - _____ Do you have any health, medical or dental plans, private, company, or public agency? |
| Registration primary care in family health teams | - | - | V0233 - Household registered at the family health unit | B001 - Is your home registered with the family health unit? |
| Self-assessment of health status | V1303 - In general, consider your own health status as: | V1303 - In general, consider your own health status as: | V1303 - In general, consider your own health status as: | J001 - In general, what is the health status of _______ |
| Limitation of usual activities in the last 15 days | V1304 - In the last two weeks, did you stop performing any of your usual activities (work, school, play, etc.) for health reasons? | V1304 - In the last two weeks, did you stop performing any of your usual activities (work, school, play, etc.) for health reasons? | V3304 - In the last two weeks, did you stop performing any of your usual activities (work, school, play, etc.) for health reasons? | J002 - In the past two weeks, did _____ stop performing any of your usual activities (working, going to school, playing, doing chores, etc.) for health reasons? |
